# A cross sectional study on lens induced glaucoma in COVID era

**DOI:** 10.6026/973206300222430

**Published:** 2026-04-30

**Authors:** Dhirendra Kumar Pandey, Aashi Jain, Praher Shrivastava, Yasha Bandil, Siddharth Jain

**Affiliations:** 1 Department of Ophthalmology, Birsa Munda Government Medical College, Shahdol, Madhya Pradesh, India; 2 Department of Ophthalmology, Shyam Shah Medical College & Associated SGMH Hospital, Rewa, Madhya Pradesh, India; 3 Department of Ophthalmology, Atal Bihari Vajpayee Government Medical College, Vidisha, Madhya Pradesh, India; 4 Department of Ophthalmology, MGM Eye Institute, Raipur, Chhattisgarh, India; 5 Department of Surgery, Shyam Shah Medical College, Rewa, Madhya Pradesh, India

**Keywords:** Secondary glaucoma, lens induced glaucoma (LIG), intraocular pressure (IOP), phacomorphic glaucoma, visual acuity (VA), neovascularglaucoma (NVG)

## Abstract

Secondary glaucoma is a diverse group of disorders characterized by elevated intraocular pressure (IOP) due to identifiable ocular or systemic causes, leading to optic nerve damage and vision loss. A prospective longitudinal hospital-based study was conducted with 70 patients of secondary glaucoma. Data shows that females outnumbered males in with phacomorphic type of Lens induced glaucoma (LIG). Further, high visual outcome was seen in LIG and low in neovascular glaucoma (NVG). Delayed presentation, often due to non-financial reasons, was linked to worse outcomes.

## Background:

Glaucoma is one of the leading causes of blindness worldwide. As reported by Tham *et al.* there are approximately 64.3 million cases of glaucoma globally, which is expected to increase to 111.8 million by 2040 [1]. In India, about 11.2 million patients aged 40 years and older and in Europe more than 12 million are affected by glaucoma and this burden is projected to grow by more than one million people by 2050 in Europe [2]. Throughout most of the 20th century, classification systems have divided glaucoma into primary and secondary forms, with developmental glaucoma as the third subset. Secondary glaucoma is not a single disease entity but rather a group of disorders in which an identifiable ocular or systemic cause results in increased intraocular pressure (IOP), leading to optic nerve damage, visual field defects and potentially vision loss if left untreated. Unlike primary glaucoma, the prevalence of secondary glaucoma is not well established due to scarcity of data, but available studies report prevalence between 6% and 22% of all glaucoma cases [2, 3- 4]. Secondary glaucoma may manifest as either open-angle or angle-closure type and can be unilateral or bilateral. Common etiologies include lens pathologies, uveal tissue inflammation, trauma, diabetes and certain drugs like steroids. It may also occur following intraocular surgeries [5].

The clinical presentation of the causative disease usually overshadows the signs and symptoms of secondary glaucoma, which may lead to delayed diagnosis and treatment [6]. Unlike primary glaucoma, individuals with secondary glaucoma often report promptly to healthcare facilities due to marked diminution of vision along with pain and ocular discomfort [7]. Therefore, it is of interest to identify the common primary pathologies causing secondary glaucomas, their clinical profile and treatment outcomes at a tertiary care center in central India.

## Methodology:

## Study design and setting:

This hospital-based prospective longitudinal study was conducted in the Department of Ophthalmology, Shyam Shah Medical College and associated GM Hospital, Rewa, India, from January 2021 to June 2022.

## Study population:

Seventy patients with secondary glaucoma attending the Department of Ophthalmology were enrolled in the study. All patients with secondary glaucoma were included, while those with primary glaucoma and developmental glaucoma were excluded.

## Data collection:

After obtaining written informed consent, data was collected from all subjects, including demographic characteristics (age, gender, residence and occupation). A detailed history was taken regarding presenting complaints, duration of symptoms, past ocular or systemic diseases, history of trauma, previous ocular surgeries and drug intake, with special reference to corticosteroid use.

## Clinical examination:

All patients underwent a comprehensive ophthalmic evaluation that included:

[1] Best-corrected visual acuity (BCVA) using Snellen's chart

[2] Examination of ocular adnexa

[3] Intraocular pressure (IOP) measurement by Applanation tonometry

[4] Pupillary reaction assessment

[5] Slit-lamp examination of the anterior segment

[6] Gonioscopy for angle evaluation

[7] Posterior segment evaluation using slit-lamp bio microscopy with Volk 90-D lens, indirect ophthalmoscope and direct ophthalmoscope

[8] B-scan ultrasonography in cases where fundus visualization was not possible

[9] Visual field examination using Humphrey Field Analyzer when feasible

[10] Spectral Domain Optical Coherence Tomography (SD-OCT) for corneal thickness, macular thickness and RNFL thickness assessment when required

## Treatment and Follow-up:

Treatment was carried out as per standard guidelines depending on the causative factors. Medical management included topical and systemic anti-glaucoma and anti-inflammatory medications. Surgical interventions were performed as needed. All patients were followed up at 1 week, 1 month and 3 months post-treatment. At each follow-up visit, a complete ophthalmic examination was conducted.

## Statistical analysis:

The collected data was analyzed using MS Excel. For demographic and clinical profile, percentage-based comparisons were made and means with standard deviations were calculated for parameters like IOP. Chi-square test was applied to assess the association between final visual outcome and duration of symptoms and Spearman rank correlation was used to evaluate the association between presenting IOP and final visual outcome.

## Results:

This table summarizes the demographic characteristics and initial clinical findings of the study population. The patients ranged from 18 to 85 years old, with a mean age of 61.08 ± 14.08 years. The most commonly affected age group was 61-80 years, comprising 67.14% of the cohort. Females slightly outnumbered males (55.71% vs. 44.29%) with a female-to-male ratio of 1.25:1. At presentation, a significant majority (91.4%) had visual acuity (VA) worse than 6/60. The mean intraocular pressure (IOP) across all patients was 37.87 mmHg, with the highest mean IOP observed in neovascular glaucoma (NVG) cases (41.57 mmHg) and followed by traumatic glaucoma (39 mmHg) (Table 1 - see PDF). This table provides a breakdown of the types and mechanisms of secondary glaucoma observed. Lens induced glaucoma (LIG) was the most prevalent form, accounting for 64.28% of cases, followed by neovascular (10%), uveitic (8.57%) and traumatic glaucoma (7.14%). Less common types included pigmentary, pseudoexfoliative glaucoma, iris cyst, raised episcleral venous pressure (EVP) and intraocular tumors. Mechanistically, 70% of cases were secondary angle closure glaucoma (SACG), while 30% were secondary open angle glaucoma (SOAG). Among SACG cases, phacomorphic glaucoma was the leading cause, responsible for over half (51.47%) (Table 2 - see PDF). This table compares the clinical features of the four major subtypes: LIG, NVG, uveitic and traumatic glaucoma. LIG patients had the highest mean age (66.21 years), with a female predominance and phacomorphic mechanism dominating. NVG patients were younger (mean age 51.33), all had very poor initial vision and none showed significant visual improvement post-treatment. Uveitic glaucoma showed a mixed mechanism (SOAG and SACG) with moderate visual recovery in half the cases. Traumatic glaucoma affected younger, mostly male patients, primarily due to hyphema and angle recession, with mixed visual outcomes and complications like vitreous hemorrhage and retinal detachment (Table 3 - see PDF). This table outlines the less common forms of secondary glaucoma. Pseudoexfoliative and pigmentary glaucomas were seen in two male patients each, with the former being bilateral and in older individuals (mean age 73), while the latter occurred in younger males (mean age 40). The remaining three cases-iris cyst, raised EVP and intraocular tumor-were singular and lacked detailed demographic or clinical data, reflecting their rarity and diverse etiologies (Table 4 - see PDF). This table presents the post-treatment visual outcomes. Over half of the patients (52.85%) achieved a VA of ≥6/18 at 3 months, most commonly among LIG patients. In contrast, 30% had poor outcomes with VA <6/60, predominantly in NVG cases. LIG, especially in patients who presented early, was associated with the best prognosis, while NVG showed the poorest outcomes despite intensive therapy, highlighting the importance of early detection and subtype-specific management strategies (Table 5 - see PDF).

## Discussion:

Secondary glaucoma presents a significant challenge in ophthalmic practice due to its diverse etiology and often poor visual outcomes. Our study of 70 patients with secondary glaucoma revealed important insights into its pattern and treatment outcomes in a tertiary care setting in central India. The mean age of patients in our study was 61.08±14.08 years, with a female preponderance (F:M ratio of 1.25:1). This finding is consistent with Chakma *et al.* who also observed a female predominance with an F: M ratio of 1.1:1 [8]. However, studies by Helayel *et al.* [9] and Gurung *et al.* [10] found males to be more affected by secondary glaucoma. The predominance of the age group 61-80 years (67.14%) in our study differs from Gurung *et al.* who reported maximum cases (40.7%) in the 40-60 year age group. Lens induced glaucoma was the most common cause (64.28%) of secondary glaucoma in our study, which aligns with findings by Gurung *et al.* [10] and Chakma *et al.* [8], who reported LIG as the most frequent cause (32.8% and 44.34%, respectively). This high prevalence of LIG in developing countries like India can be attributed to factors such as lack of access to timely cataract surgery, financial constraints and lack of awareness about the need for early intervention. In contrast, studies from developed countries show different patterns. Helayel *et al.* [9] found pseudoexfoliative glaucoma to be the most common cause (35.8%) in Saudi Arabia, while Sherpa and Pokhrel [11] reported NVG as the most common cause in their study. These variations likely reflect differences in healthcare access, socioeconomic factors and population demographics. Our study found a significant female predominance (F:M ratio of 1.6:1) among LIG patients, consistent with findings by several other researchers [12, 13-14]. This gender disparity may be explained by factors such as older females receiving less attention in some communities, financial dependence on family members and hence delayed surgical treatment for cataracts. Additionally, Sharma *et al.* [15] proposed that cataract is more common in females due increased longevity and limited healthcare access. Phacomorphic glaucoma was the most common type of LIG in our study (80%), which aligns with findings, by Sinha *et al.* [12] (72%). However, Ramanarao and Jain [4] found phacolytic glaucoma (46%) to be more common than phacomorphic (42%) in their study. We found a significant association between duration of symptoms and final visual outcome (p<0.05), with poorer outcomes among patients who presented late (≥15 days). This finding is consistent with Stuart *et al.* [2] who also reported that delayed presentation (>15 days) was strongly associated with poor visual outcomes. Interestingly, we found that non-financial factors, particularly good vision in the fellow eye and lack of escort, were the primary reasons for delayed presentation, rather than financial constraints. This differs from findings by Sinha *et al.* [12] and Seth *et al.* [14] who identified financial constraints as the main barrier to seeking early treatment. Proliferative diabetic retinopathy (57.14%) and central retinal vein occlusion (42.86%) were the main causes of NVG in our study, consistent with findings by Seth *et al.* [3] and Gurung *et al.* [10]. The poor visual prognosis of NVG observed in our study, with no significant improvement even after treatment, underscores the challenges in managing this aggressive form of secondary glaucoma. Uveitic glaucoma constituted 8.57% of cases in our study, which is higher than the 2.8% reported by Gurung *et al.* [10] but lower than findings by Liu *et al.* [16], who identified inflammatory glaucoma as the third most common cause of secondary glaucoma (20.9%). The mechanism of raised IOP was equally distributed between open-angle (50%) and angle-closure (50%) types in our study, reflecting the diverse pathophysiology of uveitic glaucoma. The male predominance (M:F ratio of 4:1) and younger age of presentation (mean age 39.6 years) among traumatic glaucoma patients in our study are consistent with findings by Hardjasasmita *et al.* [17], who reported similar demographic patterns. In our study, hyphema was the leading cause of IOP elevation (80%), Noman *et al.* [18] reported angle recession in with variable degree of angle cleavage in traumatic glaucoma patients. Pseudoexfoliative and pigmentary glaucoma were relatively uncommon in our setting (2.8% each), in contrast to some studies like Shua *et al.* [19] and Helayel *et al.* [9], who reported pseudoexfoliative glaucoma as the most common form of secondary glaucoma. This disparity likely reflects regional variations in disease prevalence and genetic factors. Our findings highlight the importance of early detection and prompt management of secondary glaucoma, particularly for LIG, which showed the best visual outcomes when treated early. The significant impact of delayed presentation on visual outcomes suggests a need for improved public awareness and outreach programs targeting at-risk populations, especially older individuals and women. The poor prognosis of NVG emphasizes the importance of early screening and management of underlying conditions like diabetes and retinal vascular diseases. Additionally, the finding that good vision in the fellow eye was a major reason for delayed presentation highlights the need for patient education about monitoring each eye individually.

## Conclusion:

Secondary glaucoma primarily affects middle-aged and elderly individuals, with a higher prevalence in females. Lens-induced glaucoma, especially phacomorphic type, was common. Early detection and intervention are critical, as delayed presentation-often due to non-financial barriers-negatively impacts outcomes. Improved awareness, timely care and addressing access challenges are essential to reduce vision loss from secondary glaucoma.
